# Supplementary material for: A qualitative study examining stressors among Respiratory Therapists in Ontario amidst the COVID-19 pandemic
Source: PLoS One. 2024 Dec 12;19(12):e0312504. doi: 10.1371/journal.pone.0312504 (PMC11637264; doi:10.1371/journal.pone.0312504)
Supplement: S1 File — (DOCX) [file pone.0312504.s001.docx]

**Respiratory Therapist Interview Guide**

1. I will first ask you demographic questions:
   1. What is your name, and what province do you represent?
   2. How old are you?
   3. I will ask you some questions, so please choose one. What is your gender?
      1. Woman
      2. Man
      3. My gender identity is not listed
      4. I choose not to respond
   4. Which of these apply to you? What is your marital status?
      1. Married
      2. Cohabitating
      3. Divorced
      4. Separated
      5. Single, never married
      6. My relationship status is not listed here
      7. I choose not to respond
   5. What is the primary location of your work (e.g., hospital, franchise, etc.) and is it considered a rural or an urban location?
   6. How many years have you been in practice? At this location?
2. Briefly discuss the primary job roles included in your position.
3. We understand that being a Respiratory Therapist can be very stressful. Take me back to before COVID-19. What was stressful about being an RT?
4. What changed for you in March 2020?
   1. What was it like throughout the pandemic – your practice? The environment? Relationships? Life at home?
   2. Tell us about the stressors you have been facing during the pandemic and specify a timeline, for instance, during the first wave.
   3. What were the implications of these stressors on patient care?
5. Tell me about stressors outside of work that might have contributed to your stress at work.
6. Looking ahead, what are some of the lessons learned from this pandemic that could help reduce stress in the event of future pandemics?
7. From the lens of your profession and your experiences working during the pandemic, how can we improve the healthcare system? In other words, what can be done to help optimize the healthcare system, even beyond the pandemic?
8. Anything you would like to add?
