# Supplementary material for: A qualitative study examining stressors among Respiratory Therapists in Ontario amidst the COVID-19 pandemic
Source: PLoS One. 2024 Dec 12;19(12):e0312504. doi: 10.1371/journal.pone.0312504 (PMC11637264; doi:10.1371/journal.pone.0312504)
Supplement: Attachment — Submitted filename: RT Reviewer responses.docx [file pone.0312504.s002.docx]

**Re: PLOS ONE Decision: Revision required [PONE-D-24-05435]**

May 26, 2024

Dear Reviewers,

We appreciate you and the reviewers for reviewing our paper (Breathing Under Pressure: Examining Stressors Among Respiratory Therapists in Ontario Amidst the COVID-19 Pandemic) and providing valuable comments. The insightful comments have led to improvements in the current version. The authors have carefully considered the comments and worked to address each in a point-by-point table below.

All the modifications are below and in the manuscript with track changes. The authors welcome further constructive comments on the clarity of our arguments, the relevance of our references, and any other aspect that could enhance the quality of our paper.

| **Comment** | **Response** |
| --- | --- |
| **Editor** |  |
| 1. Please ensure that your manuscript meets PLOS ONE's style requirements, including those for file naming. The PLOS ONE style templates can be found at  <https://journals.plos.org/plosone/s/file?id=wjVg/PLOSOne_formatting_sample_main_body.pdf> and  <https://journals.plos.org/plosone/s/file?id=ba62/PLOSOne_formatting_sample_title_authors_affiliations.pdf> | Thank you for your feedback. We have updated manuscript based on the provided resources. |
| 2. Please provide additional details regarding participant consent. In the ethics statement in the Methods and online submission information, please ensure that you have specified (1) whether consent was informed and (2) what type you obtained (for instance, written or verbal, and if verbal, how it was documented and witnessed). If your study included minors, state whether you obtained consent from parents or guardians. If the need for consent was waived by the ethics committee, please include this information. | We have clarified in the Methods section the ethics statement as follows:  Before the interview, participants were informed they had the right to refuse to answer questions and/or withdraw from the study without any consequences. All participants provided written informed consent via email before starting their interviews. |
| 3. We note that your Data Availability Statement is currently as follows: All relevant data are within the manuscript and its Supporting Information files.  Please confirm at this time whether or not your submission contains all raw data required to replicate the results of your study. Authors must share the “minimal data set” for their submission. | Yes, our submission contains all the raw data required to replicate the results. |
| 4. Please include your full ethics statement in the ‘Methods’ section of your manuscript file. In your statement, please include the full name of the IRB or ethics committee who approved or waived your study, as well as whether or not you obtained informed written or verbal consent. If consent was waived for your study, please include this information in your statement as well. | We have included the following statement:  Ethics approval was obtained from the University of Guelph’s research ethics board (REB#22-03-001, Approval date: 04 May 2022). |
| **Reviewer 1** |  |
| Reviewer #1: Saragosa et al performed a qualitative study evaluating stressors among RTs working in Canada. This is an excellent study and well-written. I have a few questions and comments below to help improve the quality of the work. Importantly, there are some missing citations from the RT literature on wellness. Lastly, are any of the authors RTs? If not, I would suggest asking an RT for further insight to sharpen the discussion with more specifics on what can be done to help the mental wellness of RTs. | Thank you for making this suggestion. None of the original authors are Respiratory Therapists. In response to your suggestion, we have had an RT review the paper to provide additional feedback and insights. This individual preferred to be acknowledge. Accordingly, we have included the following ‘Acknowledgements’ statement:  **Acknowledgements**  We acknowledge the contribution made to the review of this paper and substantive support for recruitment from Carolyn McCoy, a staff member of the Canadian Society of Respiratory Therapists. We also acknowledge the valuable contribution and stories of the respiratory therapist participants. |
| Title: Please remove Breathing Under Pressure, while RTs help people breath, the subjects of this study are the RTs themselves. Are any of the authors RTs? | We have revised the title to the following:  **A Qualitative Study Examining Stressors Among Respiratory Therapists in Ontario Amidst the COVID-19 Pandemic** |
| Abstract Background can be written more clearly. The pandemic caused massive surges in critically ill patients requiring mechanical ventilation, which overloaded RT departments and caused very high workloads. | We have simplified the beginning portion of the abstract in the follow way:  Health care systems were subjected to an unprecedented surge of critically ill patients with the coronavirus disease 2019 (COVID-19), which required management by Respiratory Therapists (RTs). |
| Manuscript Page 4, line 88 requires citation and specific examples of psychological distress. Would suggest Miller et al doi:10.4187/respcare.09283 and Strickland et al doi: 10.4187/respcare.10144 or a review article from the same group Miller et al doi: 10.4187/respcare.10632. | Thank you for bringing this oversight to our attention. We have added the suggested references.  In particular, RTs’ COVID-19 exposure and caseload may make them susceptible to higher levels of psychological distress ^1-3^. |
| Reference 22 was not a study of burnout but rather of what supportive resources were available. Would change reference to Miller et al doi:10.4187/respcare.09283. Citations are needed for pay and leadership being drivers of burnout. | We have updated reference 22 to the suggested Miller et al. paper.  We have added the Strickland et al. (DOI: <https://doi.org/10.4187/respcare.10144>) to support leadership and pay in the context of burnout. |
| Results First sentence starts with a lower case of, is there part of the sentence missing? What do you mean by 27 participants who led interviews? Does this mean participated? | Thank you for this observation. We have corrected the sentence:  Of the twenty-seven participants, twenty-one participated in a one-on-one interview, and six participated in focus groups comprising three RTs each |
| Do you think the interviewees were representative of the total population of RTs? What role did they have in their hospital? The views of front-line clinicians in the ICU will likely be different than leaders or staff less exposed to COVID-19. | No, we do not claim that the interviewees are representative of the total population of RTs. In qualitative research, we are not seeking representativeness since the qual paradigm is based on interpretivism. Instead, we are more interested in a purposeful sample of individuals who have a shared experience, which contributes to identifying patterns and shared meaningful across participants. |
| Why was this categorized as working in the shadows and suffering in silence instead of the simpler definition like (lack of appreciation or acknowledgement)? The others themes are similar, it seems the authors are trying to be clever instead of simply describing what is happening. | The authors are not trying to be clever, rather, the authors have tried to use the words expressed by the participants themselves. For example, “working in the shadows” was stated by RT001 when describing their experience as an RT.  We have clarified this to ensure that readers understand that the themes are derived from the expressed words as opposed to the authors:  The themes were constructed using the words of the participants from the text. |
| I would suggest adding some tables or figures to help more clearly present the results. | We have added two summary tables. |
| Discussion While additional qualitative research would be helpful, available quantitative surveys have identified a number of factors associated with burnout (which can be used as a marker of stress). I would suggest mentioning them, drawing on the review article from Miller et al cited above. | Thank you for your comment. Based on this and the comment below, we consulted a Registered Therapist from Ontario for review and feedback of the paper. The following citations have been added to strengthen the focus on post-pandemic systematic issues:  51. Omar AS, Hanoura S, Labib A, Kaddoura R, Rahhal A, Al-Zubi MM, et al. Burnout among respiratory therapists and perception of leadership: A cross sectional survey over eight intensive care units. Journal of Intensive Care Medicine. 2022;37(12):1553-62.  52. Kelly LA, Gee PM, Butler RJ. Impact of nurse burnout on organizational and position turnover. Nursing outlook. 2021;69(1):96-102.  56. Sangal RB, Bray A, Reid E, Ulrich A, Liebhardt B, Venkatesh AK, et al., editors. Leadership communication, stress, and burnout among frontline emergency department staff amid the COVID-19 pandemic: A mixed methods approach. Healthcare; 2021: Elsevier.  58. Canadian Society of Respiratory Therapists. Policy Recommendations for the Health Human Resource Crisis2022 May 21, 2024. Available from: <https://www.csrt.com/wp-content/uploads/Policy-Recommendations-for-the-Health-Human-Resource-Crisis_August-11-2022.pdf>.  63. Canadian Society of Respiratory Therapists, editor Proceedings from the Canadian Society of Respiratory Therapists Annual Conference. CSRT Annual Conference; 2024; Banff, Alberta, Canada: Canadian Journal of Respiratory Therapy. |
| I would reframe a lot of this discussion to address post-pandemic systemic issues such as staffing, emotional support, and other factors respiratory care departments and hospitals can do to better support RTs. |  |
| In the limitations section, your sample is actually quite small and you need to present more data (or acknowledge it is non-representative) about the respondents. | We disagree that our sample is small. We are also not seeking representativeness of RTs since that can only be done using probability sampling methods.  In previous RT qualitative research sample sizes were less:  <https://openurl.ebsco.com/EPDB%3Agcd%3A12%3A3001580/detailv2?sid=ebsco%3Aplink%3Ascholar&id=ebsco%3Agcd%3A138860046&crl=c>  <https://www.ncbi.nlm.nih.gov/pmc/articles/PMC10769513/> (this study had 15 RTs from Ontario)  <https://www.ncbi.nlm.nih.gov/pmc/articles/PMC9350338/> |
| Reviewer 2 |  |
| Were the interviews conducted by one team member or more than one? How were the interviewers trained to reduce variability among the individual interviews/focus groups? | We clarified the number of interviewers in the following sentence:  The interviews were conducted by one member of the research team virtually using |
| I assume that you used traditional text coding (versus electronic coding software) but that isn't explicitly stated. | We indicated in the following sentence that the coding happened manually with Microsoft Word as opposed to coding software:  All data analyses were performed manually using Microsoft Word 365 on Mac. (Page 7) |
| Would like to see the actual interview questions included in the manuscript as a supplementary document. | We have included the interview guide as a supplementary document. |
| Line 118: I think you mean "formerly known as Twitter" (not formally) | Thank you for noticing this error. We have revised to say formerly. |
| Line 150: Is there a word missing at the beginning of the paragraph or is the "O" in "of" supposed to be capitalized? | Yes we noticed that the sentence must have been caught off. We have corrected the sentence. |
| Line 306 - not sure why anxiety is in quotations. | It was put in quotes because anxiety came directly from the participants, as opposed to the authors interpreting the experience of anxiety. We removed the quotation marks to avoid confusion. |
| Line 312-313 - looks like sentence fragment | We have revised the statement to address sentence fragmentation:  In the summers since COVID-19, RSV and influenza on top of COVID replaced lower patient volumes |

Sincerely,

**Marianne Saragosa, RN PhD**

Embedded Nurse Scientist

Science of Care Institute

Sinai Health, Toronto, ON
